# The weird eusociality of polyembryonic parasites

**DOI:** 10.1098/rsbl.2021.0026

**Published:** 2021-04-07

**Authors:** Brian A. Whyte

**Affiliations:** Department of Environmental Science, Policy and Management, University of California, Berkeley, CA, USA

**Keywords:** eusociality, polyembryony, soldier castes, social evolution, trematodes, parasites

## Abstract

Some parasitoid wasps possess soldier castes during their parasitic larval stage, but are often neglected from our evolutionary theories explaining caste systems in animal societies. This is primarily due to the polyembryonic origin of their societies. However, recent discoveries of polyembryonic trematodes (i.e. flatworms) possessing soldier castes require us to reconsider this reasoning. I argue we can benefit from including these polyembryonic parasites in eusocial discussions, for polyembryony and parasitism are taxonomically vast and influence the evolution of social behaviours and caste systems in various circumstances. Despite their polyembryony, their social evolution can be explained by theories of eusociality designed for parent–offspring groups, which are the subjects of most social evolution research. Including polyembryonic parasites in these theories follows the trend of major evolutionary transitions theory expanding social evolution research into all levels of biological organization. In addition, these continued discoveries of caste systems in parasites suggest social evolution may be more relevant to parasitology than currently acknowledged.

## 1. The new eusocial systems

Eusociality is one of the most substantial guiding paradigms for social evolutionary research. Since its popularization in the mid-twentieth century [1], researchers across study taxa and disciplines have engaged in a shared evolutionary theory for how animals evolve overlapping generations, cooperative brood care and reproductive division of labour. Originally referring to certain species of Hymenoptera (e.g. ants, bees, wasps) and Isoptera (termites), the eusocial category has expanded to include species of aphids [2], thrips [3], shrimps [4], beetles [5] and naked mole rats [6]. Recently, however, larval colonies of trematodes (i.e. flatworms, blood flukes) are argued to be eusocial, following the discovery of morphologically distinct soldier castes [7], and this claim has received growing support [8–13]. This discovery is unexpected and exciting, extending our social evolutionary theories into a phylum (Platyhelminthes) that seemingly had no relevance to social evolution research. It is very confusing, therefore, that this phenomenon of soldier larvae in a parasitic colony has been known in polyembryonic wasps since 1981 [14], but is still often rejected as an example of eusociality [14–18], and even neglected from otherwise broad discussions of social evolution in wasps and Hymenoptera [19–23].

Polyembryonic wasps have been rejected from the category of eusociality because of their lack of overlapping parent–offspring generations, which is a requirement of the still popular *sensu-*Wilson definition [1]. New definitions for eusociality have been proposed by multiple authors since the 1990s, and virtually all of them pull importance away from overlapping generations, focusing more attention on comparing taxa by their reproductive divisions of labour [24–27]. Unfortunately, updating the deep terminology of social evolution research has been slow and controversial [28,29], and arguably no new consensus has been reached [30]. The eusocial status of polyembryonic wasps remained unique and uncertain until recently. Trematodes are also polyembryonic parasites with soldier castes in their larval colonies, but they technically do possess overlapping generations [31]. To consider only one of these systems as eusocial is confusing and contradictory—a case of semantics clouding comparative biology.

Overcoming this comparative confusion requires more than a terminological debate. Overlapping parent–offspring generations remain important because parental care (i.e. subsociality) is a firmly established prerequisite in evolutionary theories of sterile castes in animal societies [32,33]. Beyond semantics, how do we account for larval colonies of parasites that have converged upon sterile helper castes absent of the family-living context we observe in all other eusocial systems? Answering this is important, as polyembryonic parasites are useful exceptions to the eusocial norms receiving much attention from research on reproductive divisions of labour. Indeed, parasites are potentially full of undiscovered systems possessing behaviours convergent to social and eusocial taxa [34–37], and if we are going to include this incredibly common lifestyle in our social evolutionary theories, we can start by understanding the weird eusociality found in polyembryonic parasites.

## 2. Polyembryonic soldier castes do not require overlapping generations

**Figure 1.**
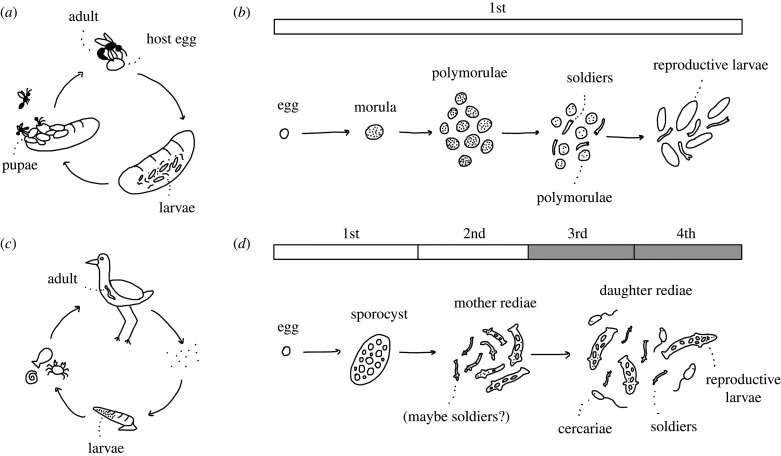
Life cycle and larval development in polyembryonic wasps and trematodes. (*a*) Polyembryonic wasps (e.g. *Copidosoma floridanum* [38,39]) lay one or more eggs into hosts, which develop into larvae and pupae while in this host. (*b*) The egg becomes a morula, splitting into polymorulae, which develop into sterile soldiers or regular larvae (i.e. ‘reproductive larvae’) that pupate and become sexually mature. All of these developmental stages are technically one generation (white bar). (*c*) Trematode (e.g. *Himasthla rhigedana* [40–42]) adults lay eggs which are released from their vertebrate hosts, find snails and multiply into a population of larvae. (*d*) Trematode eggs develop into a single sporocyst larva, which produces the first generation of rediae (i.e. larvae with mouths). It is unknown if soldier morphs are also produced in this first generation, but soldiers are certainly present in the daughter rediae generation, as well as cercariae—the dispersive morph. Multiple generations overlap during the daughter generations (grey shaded bar).

Trematode and polyembryonic wasp species with soldier castes share similar life histories (figure 1) and selective pressures. In each case, an endoparasitic population originates from polyembryony, where a single egg splits into multiple embryos [38,43] and some of these embryos become morphologically and behaviourally distinct soldiers, improving the fitness of their colony by attacking competitors developing in the same host [44,45]. The single difference leading trematodes to be called eusocial, but not polyembryonic, wasps is that the first generation of trematode larvae descending from polyembryony continue to asexually produce new generations of larvae, while the polyembryonic wasp larvae do not (figure 1*b*,*d*). For these parasites, these overlapping generations only highlight differences in polyembryonic development. Unlike for the bees in which eusociality was first described [46], the presence or absence of overlapping generations in these parasites does not determine what brood care behaviours or reproductive divisions are capable of evolving. Importantly, ‘overlapping generations’ in most contexts refers to sexually mature stages of a life cycle spatially associated with offspring in earlier life stages, but this is never the case for polyembryonic parasites.

Trematodes are similar to the gall-forming aphids which can exhibit all the criteria of eusociality only during their asexual multiplication stage inside galls [2,21]. In trematodes and aphids, a multigenerational population can occur during these asexual life stages, allowing kin to specialize in caring for their developing siblings. In polyembryonic wasps, a ‘multi-developmental’ population occurs, where the polymorulae descending from the same zygote develop at different rates on different pathways [38,47], with soldiers developing before their siblings become larvae or pupae. This allows a caste system and brood caring relationship to form within one short-lived generation, absent of parents, as brood care for each other.

This quality of non-helpless brood is expected for parasite life cycles that use multiple hosts, or only use hosts for certain life stages, since each life stage might develop isolated from the previous one, effectively preventing direct care across generations. It is also the norm for some termites, aphids and other hemimetabolous social insects where the brood are precocial and perform tasks as juveniles [19,48–50]. Despite their differences, polyembryonic parasites have converged upon a hallmark of other eusocial taxa (soldier castes), and they do not represent an alternative explanation for eusociality. If we look past their lack of overlapping generations or traditional parental care, these polyembryonic groups fit surprisingly well into modern theories explaining eusociality in animal groups with overlapping generations.

## 3. Family living facilitates social evolution, but so does polyembryony

Why are overlapping generations important for the evolution of eusocial groups? While convincing arguments have been made for why we can ignore this trait in our terminological categories [25], parent–offspring grouping plays a fundamental role in the evolution of social behaviours via kin selection [51], and is incorporated into many theoretical frameworks of social evolution [52–56]. However, family living is not intrinsically important. It is the benefits and the consequences of family living that make it relevant to our evolutionary theories [57]. Family living is a proxy for more specific traits that facilitate social evolution, and polyembryonic groups achieve many of these same qualities (table 1). For instance, family groups and clonal groups achieve high relatedness facilitating the evolution of cooperative or altruistic behaviours following Hamilton's rule [51]. The resilience and relevance of Hamilton's theories have contributed to the popularity of the subsocial (i.e. fraternal) hypothesis for evolving eusociality [20,58,59], countering the alternative semi-social (i.e. egalitarian) hypothesis [54]. Interestingly, even controversial alternatives rejecting Hamilton's rule [60] suggest group living inside a shared food source can substitute for a family living or kinship requirement. All endoparasites live inside their food source, and most, if not all, polyembryonic parasites are endoparasitic [17].

**Table 1.** Similarities and differences between social groups featuring parent–offspring overlap (i.e. family living) versus larval colonies descending from polyembryony.

characteristics	family living	polyembryony
spatial and temporal overlap of individuals	✓ yes, living in the same nest	✓ yes, living in the same host
high genetic relatedness	✓ 50, 75 or 100% related	✓ 100% related
variety of developmental stages	✓ yes, owing to production of multiple generations	✓ yes, owing to embryos developing at different rates (polyembryonic wasps) or larvae asexually reproducing (trematodes)
offspring help other offspring	✓ foraging for non-self, nest defence, reproductive sacrifice	✓ nest defence, reproductive sacrifice
offspring help developing young	✓ adults care for and/or defend brood	✓ brood defend brood, even within the same generation
offspring help parents	✓ adult offspring care for and/or defend mother	× mother is absent; soldiers defend brood in her absence

An extension of the subsocial route to eusociality is the ‘lifetime monogamy’ hypothesis, which predicts that the ancestral state of all eusocial groups with obligate sterile helpers was once both subsocial and monogamous [55]. A central argument for the importance of this bottleneck origin is that the offspring of a monogamous pair are the closest a sexually produced group of animals can come to having a shared singular origin analogous to the zygotes of multicellular eukaryotic organisms. A polyembryonic colony is even more similar to this, being a group of animals literally descending from a single egg.

While these theories emphasize the role of kinship, others emphasize ecological conditions favouring sterile helper evolution. The ‘completely overlapping generations rule’ [33] suggests that obligately sterile helpers can only evolve if they can commit their entire lifetime to raising their parent's brood. This commitment is made possible by living with a mother that lives longer than her offspring. In theory, a sterile helper can continue this commitment even if the mother is absent, as long as her brood persist and need care [33]. This is precisely the situation of polyembryonic parasites. For both trematodes and polyembryonic wasps, the mother of the polyembryonic egg is absent, but soldier morphs can spend their entire lives defending her offspring, never dispersing from their host. However, while polyembryonic wasps soldiers are sterile [38], the totipotency of trematode soldiers is not yet ruled out.

Polyembryonic parasites are consistent with core principles meant for explaining eusocial groups of parents living with adult offspring. They join other parasite taxa (aphids, thrips) as examples of ‘fortress-defender’ or ‘soldier-first’ eusociality, in which a primary function of the non-reproducing caste is nest defence, rather than foraging, feeding or housekeeping [15,19,50]. For this reason, it makes sense that authors do not include polyembryonic wasps in reviews of hymenopteran sociality [20–23], as virtually all ants, bees and wasps fit a ‘life-insurer’ or ‘worker-first’ pathway to eusociality [15,19], and parasitoid wasps are phylogenetically distinct from other social wasps.

## 4. Where do the polyembryonic parasites fit in?

Fortunately, researchers of polyembryonic wasps are aware of their similarities to eusocial taxa, and study topics such as caste determination [38,60], caste allocation [61,62], nest-mate recognition [63,64] and even sex-ratio conflict like in other Hymenoptera [65–67]. While some authors claim they are eusocial [25,68,69], others avoid explicit attribution of eusociality to polyembryonic wasps [8,41,60–62,67,70–72], or clearly state they are not eusocial [14–17] (electronic supplementary material, table S1). I urge authors to not feel pressured to fit their system into the *sensu*-Wilson definition [1], for they can cite the *sensu*-Crespi definition [25], and/or refer to them as fortress defenders [19,50], as this term was inspired by the discovery of parasites with soldier castes. The contributions from polyembryonic wasps to the evolution of reproductive division of labour are not invalidated by their lack of overlapping generations, and this character requirement was only popularized as an initial demarcation to guide, not blind, our search for eusociality across taxa [1].

How we categorize and compare eusociality in polyembryonic wasps and trematodes will change as we learn more about each system, and incorporate other instances of polyembryony and parasitism featuring divisions of labour [73]. Cnidarians and bryozoans are both polyembryonic, but can exhibit division of labour among their polyps and zooids separate from a parasitic context [74,75]. Additionally, while defence against competitors or predators is a common function of polyembryonic castes, it is not their only function (e.g. nutrient transfer in bryozoans [76], sex-ratio optimization in polyembryonic wasps [65,66]). At the moment, trematodes might be the only taxon without a clear alternative function for their soldier castes. Hypotheses on their caste evolution can be informed with the continued research on sociality in trematodes, and a better understanding of their phylogenetic relationships. For instance, trematode species exhibiting a specialized role of the first reproductive larva [77] could be viewed as a eusocial precursor, analogous to the dwarf eldest daughter in carpenter bees [78], depending on how we build our phylogenies.

Parasitism, regardless of polyembryony, can facilitate the coincidence of food, shelter and group living [35,50], which are factors relevant to social evolution in all taxa. Unique to parasitism and other host–symbiont relationships, though, is the potential influence of the host on social evolution. In aphids and thrips, soldiers are associated with host plants that prolong gall formation [21,79], but comparable metrics are not yet supported in polyembryonic wasps or trematodes. In theory, when parasite niches overlap, the fitness benefits of aggressive interference (and thus soldier morphs) should positively correlate with host characteristics that increase susceptibility (or exposure [13]) to parasite co-infections. We will learn more about the selective conditions favouring soldier castes with further understanding of parasite competitive ecology, which, serendipitously, is also a potentially useful avenue of research for medically relevant parasitology [80,81].

Beyond comparing competitive contexts, the developmental biology of these parasites is also necessary for understanding soldier caste evolution. The detailed ontogeny of polyembryonic wasps shows how the sterility of soldier morphs is determined early in development [38], but a similar depth of caste determination has yet to be described in trematode species with soldier castes.

## 5. Conclusion

Both trematodes and polyembryonic wasps possessing soldier castes can be considered eusocial, regardless of an overlapping generations criterion. Polyembryonic parasites have many differences from parent–offspring groups, but also possess many similarities that facilitate the convergent evolution of social behaviours and sterile castes. Polyembryonic parasites support the bottleneck origin of the lifetime monogamy hypothesis [55], meet the special exceptions to the completely overlapping generations rule for evolving sterile castes [33], and are more comparable to a subsocial than the semi-social route to eusociality [54]. This highlights how important relatedness and ecological conditions are for social evolutionary explanations in any system. While trematodes possess overlapping generations of larvae, and polyembryonic wasps do not, at the moment this only amounts to a difference in polyembryonic development and caste determination.

Acknowledging the eusociality of polyembryonic parasites will build a constructive conversation around the special case of polyembryony for major evolutionary transitions theories [53,82]. An egg developing on a path towards one multicellular body, eventually splitting into multiple multicellular bodies, provides unique challenges to our concepts of biological individuality. For instance, a group of polyembryonic parasites could be considered a ‘modular organism’ [18], like clonal plants or siphonophores. However, polyembryonic wasps separate germ and soma early on during embryogenesis, and the soma never exhibits modular reproduction, as occurs in other modular organisms [38]. In fact, the caste determination mechanism of polyembryonic wasps is perhaps their most fascinating contribution. Soldiers are polymorulae that never receive germinal cells [17,38]. Their separation of castes is not functionally similar to a multicellular germ/soma separation: it *is literally the same mechanism* of embryogenic cell differentiation. For this reason, polyembryonic wasps represent one of the greatest empirical confirmations of major evolutionary transitions theory, and the universal nature of social evolutionary principles across levels of biological organization.
